# Workplace Ostracism and Counterproductive Work Behaviors: The Chain Mediating Role of Anger and Turnover Intention

**DOI:** 10.3389/fpsyg.2021.761560

**Published:** 2021-12-08

**Authors:** Yingge Zhu, Denghao Zhang

**Affiliations:** Department of Psychology, Faculty of Science and Technology, Renmin University of China, Beijing, China

**Keywords:** workplace ostracism, counterproductive work behaviors, anger, turnover intention, employees born after 1990

## Abstract

This study aims to explore the mediating effect of anger and turnover intention on the relationship between workplace ostracism and counterproductive work behaviors. A two-stage follow-up survey of 426 employees born after 1990 was conducted using the Workplace Ostracism Scale, Counterproductive Work Behaviors Scale, Trait Anger subscale of the State-Trait Anger Expression Inventory, and Turnover Intention Scale. Workplace ostracism was found to be significantly positively correlated with anger, turnover intention, and counterproductive work behaviors. Furthermore, anger and turnover intention both separately and serially mediated the relationship between workplace ostracism and counterproductive work behaviors. This study confirms the chain mediating effect of anger and turnover intention on the relationship between workplace ostracism and counterproductive work behaviors.

## Introduction

*Counterproductive work behaviors* refer to intentionally aggressive behaviors carried out by employees that are potentially harmful to the interests of an organization or its stakeholders (Spector et al., 2006). Such behaviors can not only cause huge losses from an organization (Bolton et al., 2010) but also may cause irreparable consequences for society (Ciampa et al., 2021). Currently, research on counterproductive work behavior is common. However, the majority of participants in such studies include general employees (Meisler et al., 2020) or specific occupational groups (Filipkowski and Derbis, 2020). Research on employees’ counterproductive work behaviors from the perspective of young workforce, on the other hand, is relatively limited. According to the social change theory, society is a complex and dynamic system. To adapt to changing developmental needs, a social system must adjust and reorganize its structural relationships (Li et al., 2019b). This reorganization may have a significant impact on individuals’ values, preferences, and behaviors (Chou, 2012). Compared with employees born in the 60, 70, and 80s, the values of the young employees born in the 90s are more distinct and unique (Liao and Chen, 2017; Zhang et al., 2019c), and these values will have an impact on their cognition and behaviors (Crampton and Hodge, 2009). Therefore, exploring the attitudes and behaviors of young employees, who are becoming the backbone of many companies, is crucial for the development of companies and society. In this study, the employees born in the 90s will be used as participants in an exploration of the development of counterproductive work behaviors.

The General Aggression Model (GAM) posits that individual and situational factors affect the generation of aggressive behaviors (Anderson and Bushman, 2002). Counterproductive work behaviors, which are a type of aggressive behavior (Li et al., 2020a), are also affected by individual (Zhang et al., 2019b) and situational factors (Ma et al., 2019). Due to the relative stability of personal factors, situations, and especially ostracism – being excluded and ignored by others – one of the most common negative situations in interpersonal communication (Anderson and Bushman, 2018), its type and specific circumstances at the time of occurrence may have a greater impact on the subsequent internal state and behavioral results (Shao and Zhang, in press). To date, several theories have been applied in the Chinese context to unpack the negative effects of ostracism. According to the work-family spillover model, Liu et al. (2013a) revealed the positive effects of workplace ostracism on work-family conflict. Based on social identity theory, Wu et al. (2016) showed that workplace ostracism undermines organizational citizenship behavior. In addition, an extant dynamic componential perspective suggests that the destructive effects of workplace ostracism on creativity (Kwan et al., 2018). Recently, Xu et al. (2020), using a social exchange perspective, proposed that the negative effects of workplace ostracism on work engagement. Furthermore, the results of a meta-analysis of 95 independent samples (*N*=26,767) from Asia or the Occident revealed that exposure to workplace ostracism is significantly and negatively related to organizational identification, organizational commitment, belongingness, job satisfaction, job performance, organizational citizenship behavior, and positively related to turnover intentions, emotional exhaustion, organizational deviance, and interpersonal deviance (Li et al., 2021). Therefore, it is vital to explore the antecedent variables of employees’ counterproductive work behaviors from the perspective of ostracism in the workplace. Workplace ostracism may place individuals into marginal positions, and has the potential to cause both physical and mental pain (Liu et al., 2013b). Employees in this position may make up for their psychological losses through counterproductive work behaviors. Therefore, this study assumes that increased workplace ostracism will lead to a greater tendency to produce counterproductive work behaviors.

Although counterproductive work behaviors typically stem from stressful situations in the workplace, these situations are external inducements of counterproductive work behaviors (Lin et al., 2010), and their impact on such behaviors can be better understood by focusing on changing the internal state of individuals (Yan et al., 2014). Ostracism is a significant source of stress (Kothgassner et al., 2021), and individual emotional changes after experiencing ostracism indirectly affect aggressive behaviors (Shao and Zhang, in press). Negative emotions make it easier for individuals to immerse themselves in a state of ostracism (Chen et al., 2020), and they are more inclined to implement counterproductive work behaviors when in this state (Baka, 2015). In particular, anger – one of the few negative emotions in work situations that can make employees respond – shapes employees’ cognition, emotional states, and behaviors (Zhang et al., 2019a). Studies have shown that workplace ostracism can affect an individual’s behavior through the mediating effect of anger (Wang et al., 2020). Trait anger also plays an important role in workplace stressors and counterproductive work behaviors (Ilie et al., 2012). Therefore, this research hypothesizes that anger plays a mediating role in the relationship between workplace ostracism and counterproductive work behaviors (i.e., workplace ostracism can induce counterproductive work behaviors by influencing individuals’ anger).

The changes in an individual’s internal state after encountering ostracism can be unconscious, such as changes in emotion, which may be affected by internal tendencies such as self-protection (Ren et al., 2016). Previous studies have shown that turnover intention is a withdrawal reaction caused by self-protection when individuals are ostracized in the workplace (Li et al., 2019a). There is a significant positive correlation between workplace ostracism and turnover intention (Yin and Liu, 2013). Zhang et al. (2015) used a questionnaire survey to investigate the impact of workplace ostracism on the turnover intention of new generation migrant workers in a Chinese organization. They found that workplace ostracism had a significant positive effect on the turnover intention of this group. Therefore, workplace ostracism may cause employees who are trapped in this dilemma to consider leaving their jobs. Simultaneously, an individual’s intrinsic tendency, as an intrinsic motivation, guides their behaviors (Ajzen, 1991). When employees know that they are about to leave an organization, their sense of identity as members of the organization is reduced. They no longer pin their career development on the organization (Zhang et al., 2018a); thus, they are more likely to exhibit negative behavior, intentional absenteeism, and other counterproductive work behaviors. Previous studies have also indicated that informal job-seeking behaviors often occur before leaving a job (Porter et al., 2019). Employees who are planning to leave their jobs are more likely to spend working hours before leaving the job engaging in counterproductive work behaviors, such as searching for job information and answering job-seeking phone calls. Therefore, this study assumes that turnover intention plays an intermediary role in the relationship between workplace ostracism and counterproductive work behaviors (i.e., workplace ostracism can induce counterproductive work behaviors by influencing individual turnover intention).

Research has shown that emotions influence people’s behaviors, along with other mediation processes (Wang et al., 2012). Affective events theory posits that emotions can not only directly affect employees’ behaviors and produce emotion-driven behaviors but also indirectly affect behaviors and produce attitude-driven behaviors (Weiss and Cropanzano, 1996). According to this theory, emotional events will first trigger employees’ emotional experiences, and with the accumulation of emotions, these events will further affect employees’ work attitudes, eventually driving their behaviors (Fu et al., 2012). As mentioned earlier, workplace ostracism may make the excluded people angry, and these angry feelings may make an individual want to leave the organization, leading to the occurrence of counterproductive work behaviors. Related studies show that anger is an important trigger for employees’ turnover intention, and anger regarding negative events predicts individual turnover intention (Harlos, 2010). Therefore, we assume that anger and turnover intention play a chain intermediary role between workplace ostracism and counterproductive work behaviors (i.e., excluded employees experience more anger and turnover intention in the workplace than other employees, ultimately leading to more counterproductive work behaviors).

**Figure 1:**
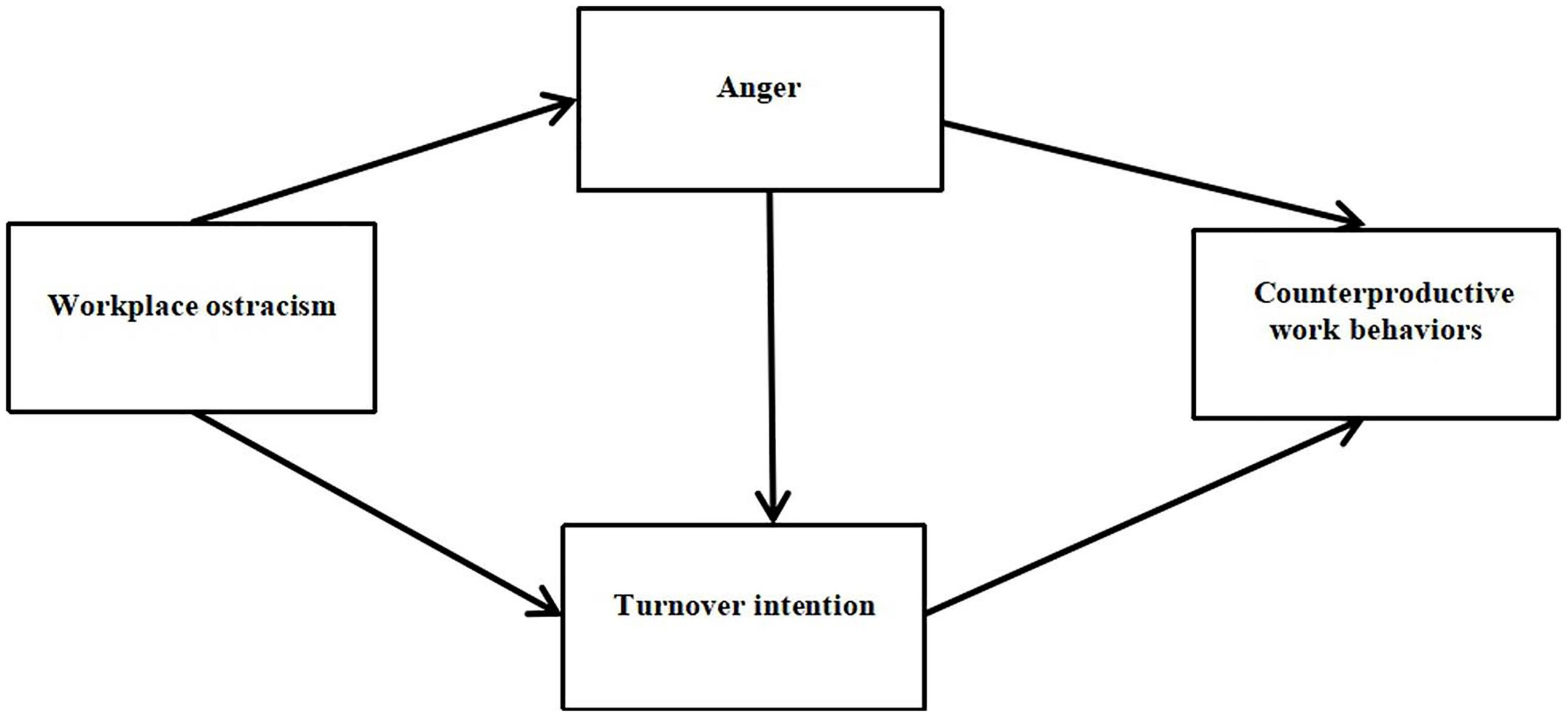
Hypothetical model diagram.

This study aims to use a questionnaire – with workplace ostracism as the independent variable, counterproductive work behaviors as the dependent variable, and anger and turnover intention as intermediary variables – to investigate the relationship between workplace ostracism and counterproductive work behaviors in young employees born after 1990. The hypothetical model is shown in Figure 1.

## Materials and Methods

### Participants and Procedure

We invited employees from different industries and companies in Shanghai and Suzhou to complete a voluntary survey. Of the 450 employees initially invited to participate in the survey, 426 usable surveys remained after matching responses and eliminating invalid questionnaires. The full survey’s effective response rate was 94.67%. In our sample, 426 employees (182 men and 244 women) completed the study. Participants’ ages ranged from 22 to 31years, including 67 (15.73%) employees aged 22–26years and 359 (84.27%) employees aged 27–31years. In terms of their highest level of education, 37 employees (8.69%) had only completed high school or less than high school, 105 employees (24.65%) had completed up to junior college, 227 employees (53.29%) had completed up to a bachelor’s degree, and 57 employees (13.38%) had completed a post-graduate degree or more. In terms of position, 314 employees (73.71%) were general workers, 76 employees (17.84%) were first-line managers, 29 employees (6.81%) were middle managers, and seven employees (1.64%) were top managers. Around 79 employees (18.54%) had worked at their company for less than 1year, 133 employees (31.22%) had worked at their company for 1–3years, 122 employees (28.64%) had worked at their company for 3–5years, and 92 employees (21.60%) had worked at their company for more than 5years.

We referred to previous research (e.g., Liu et al., 2015; Yan et al., 2020) and designed a two-wave measurement protocol at intervals of 2months to avoid common method biases (Podsakoff et al., 2003). During the first collection period, we collected information on participants’ perceived levels of workplace ostracism, levels of anger, and demographic information. During the second collection period, we collected information regarding participants’ turnover intention and counterproductive work behaviors.

Each time participants completed a questionnaire, we recorded their phone numbers, which were used as a label to match the two-wave data. Notably, before distributing the questionnaire, we informed the participants of the survey procedure, explained to all participants, and guaranteed that the survey was voluntary, confidential, and irrelevant to their performance evaluation. To reduce social desirability, we reminded the participants of the importance of answering honestly for the sake of our academic research. In addition, to motivate them for their participation, participants who completed the whole surveys were given some rewards.

### Measures

#### Workplace Ostracism

The Workplace Ostracism Scale developed by Ferris et al. (2008) has been widely used by Chinese scholars (e.g., Yan et al., 2014; Wang et al., 2020; Jiang and Zhang, 2021). It consists of 10 items, and each item is rated on a five-point Likert scale (1=*never* to 5=*always*). Participants are asked to rate each statement, for example, “others ignored you at work,” based on how often they feel the sentiment. Higher scores indicate a stronger sense of workplace ostracism. In this study, the Cronbach’s alpha for this scale was 0.94.

#### Counterproductive Work Behaviors

The Counterproductive Work Behavior Scale was originally developed by Dalal et al. (2009) and verified and revised by Chinese scholars (Liu, 2011) to align with Chinese organizations. It consists of 12 items, each rated on a five-point Likert scale (1=*never* to 5=*always*). Participants are asked to rate each statement, for example, “spent time on tasks unrelated to work,” based on how often they do the listed behavior. Higher scores indicate a higher frequency of employees engaging in counterproductive work behaviors. In this study, the Cronbach’s alpha for this scale was 0.95.

#### Anger

The Trait Anger subscale of the State-Trait Anger Expression Inventory developed by Spielberger (1999) has been widely used by Chinese scholars (e.g., Li et al., 2020b; Li and Xia, 2021). It consists of 10 items, each rated on a four-point Likert scale (1=*very non-conforming* to 4=*very conforming*). Participants are asked to rate each statement, for example, “I am very impatient,” based on how much they identify with it. Higher scores indicate higher levels of trait anger. In this study, the Cronbach’s alpha for this scale was 0.90.

#### Turnover Intention

The Turnover Intention Scale was developed by Mobley et al. (1978) and has been widely used by Chinese scholars (e.g., Xiong et al., 2015; Cheng and Lin, 2017). It consists of four items, each rated on a five-point Likert scale (1=*completely disagree* to 5=*completely agree*). Participants are asked to rate each statement, for example, “I often think about quitting my present job,” based on how much they agree or disagree with it. Higher scores indicate higher employee turnover intention. In this study, the Cronbach’s alpha for this scale was 0.90.

#### Control Variables

Accounting for the heterogeneity of the sample, we controlled for five demographic variables (i.e., gender, age, education, position, and working time) that previous studies have suggested might affect employees’ counterproductive work behaviors (e.g., Chen et al., 2017; Yan et al., 2020). Gender was measured as a dichotomous variable, coded as “1” for male and “2” for female. To protect the privacy of participants (Chen et al., 2017), age was also measured as a dichotomous variable expressed by the participants’ dates of birth (1=*1990–1995*, 2=*1996–1999*). Education was measured on a four-point scale based on the highest level of education completed by each participant (1=*high school or below*, 2=*junior college*, 3=*bachelor’s degree*, and 4=*master’s degree or above*). To avoid the potential effects of position (Chen et al., 2017), position was measured on a four-point scale (1=*general worker*, 2=*first-line manager*, 3=*middle manager*, and 4=*senior manager*). Finally, working time was measured on a four-point scale (1=*less than 1year*, 2=*1–3years*, 3=*3–5years*, and 4=*more than 5years*).

### Common Method Bias

Exploratory factor analysis was used to examine the common method bias (Zhou and Long, 2004). The results showed that the explanation rate of variance of the extracted maximal factor was 33.08%, which was far below the critical criterion of 40%. This showed that the common method bias in this study was acceptable and would not seriously affect the results of the data analysis.

## Results

### Confirmatory Factor Analysis

Prior to testing specific hypotheses, we conducted confirmatory factor analyses (CFAs) on the four self-reported scales. We first examined the fit of a four-factor model that included workplace ostracism, anger, turnover intention, and counterproductive work behaviors. As expected, the proposed four-factor model demonstrated acceptable fit [*χ^2^*(568)=1,504.98, *p*<0.001; *CFI*=0.92; *TLI*=0.91; *RMSEA*=0.06]. In addition, all factor loadings were significant, demonstrating convergent validity. The discriminant validity of the four constructs was then tested by contrasting the four-factor model against two alternative models: a three-factor model and a one-factor model. The three-factor model was obtained by loading those items measuring anger and counterproductive work behaviors into one latent factor, since among the four constructs these two had the highest correlation. The one-factor model was obtained by loading all items of the four proposed constructs into one latent factor. CFA results suggested that the three- and the one-factor models yielded poor fits to the data: *χ^2^*(591)=3,557.58, *p*<0.001; *CFI*=0.74; *TLI*=0.72; *RMSEA*=0.11 and *χ^2^*(594)=6,956.49, *p*<0.001; *CFI*=0.43; *TLI*=0.40; *RMSEA*=0.16, respectively. Therefore, the discriminant validity of the constructs used in this study was confirmed.

### Descriptive Statistics and Correlation Analysis

Table 1 displays descriptive statistics and the correlations between the study variables. As expected, the core study variables were significantly associated with each other. More specifically, workplace ostracism was positively related to anger (*r*=0.32, *p*<0.001), turnover intention (*r*=0.25, *p*<0.001), and counterproductive work behaviors (*r*=0.28, *p*<0.001). Anger was positively correlated with turnover intention (*r*=0.29, *p*<0.001) and counterproductive work behaviors (*r*=0.49, *p*<0.001). In addition, turnover intention was positively associated with counterproductive work behaviors (*r*=0.40, *p*<0.001).

**Table 1:** Descriptive statistics and correlations among all variables.

	*M*±*SD*	1	2	3	4	5	6	7	8
1 Gender	1.57±0.50	1							
2 Age	1.16±0.36	0.13^*^							
3 Educational level	2.71±0.80	0.08	−0.14^**^						
4 Position level	1.36±0.68	−0.10^*^	−0.01	−0.03					
5 Working time	2.53±1.03	−0.14^**^	−0.40^***^	−0.17^***^	0.25^***^				
6 Workplace ostracism	1.70±0.67	−0.16^**^	0.04	−0.13^**^	0.06	0.03			
7 Counterproductive work behaviors	2.22±0.92	−0.13^**^	0.003	−0.08	0.12^*^	0.12^*^	0.28^***^		
8 Anger	2.14±0.61	−0.08	−0.04	−0.01	0.12^*^	0.13^**^	0.32^***^	0.49^***^	
9 Turnover intention	2.78±1.00	−0.05	−0.06	−0.07	−0.04	0.08	0.25^***^	0.40^***^	0.29^***^

*N*=426; (1) Gender: 1=male, 2=female; (2) Age: 1=date of birth between 1990 and 1995, 2=date of birth between 1996 and 1999; (3) Educational level (highest level of education achieved): 1=high school or below, 2=junior college, 3=bachelor’s degree, and 4=master’s degree or above; (4) Position level: 1=general worker, 2=first-line manager, 3=middle manager, and 4=senior manager; (5) Working time (Time working at organization): 1=less than 1year, 2=1–3years, 3=3–5years, and 4=more than 5years.

* *p*<0.05;

** *p*<0.01 and

*** *p*<0.001.

### Mediation Analysis

**Figure 2:**
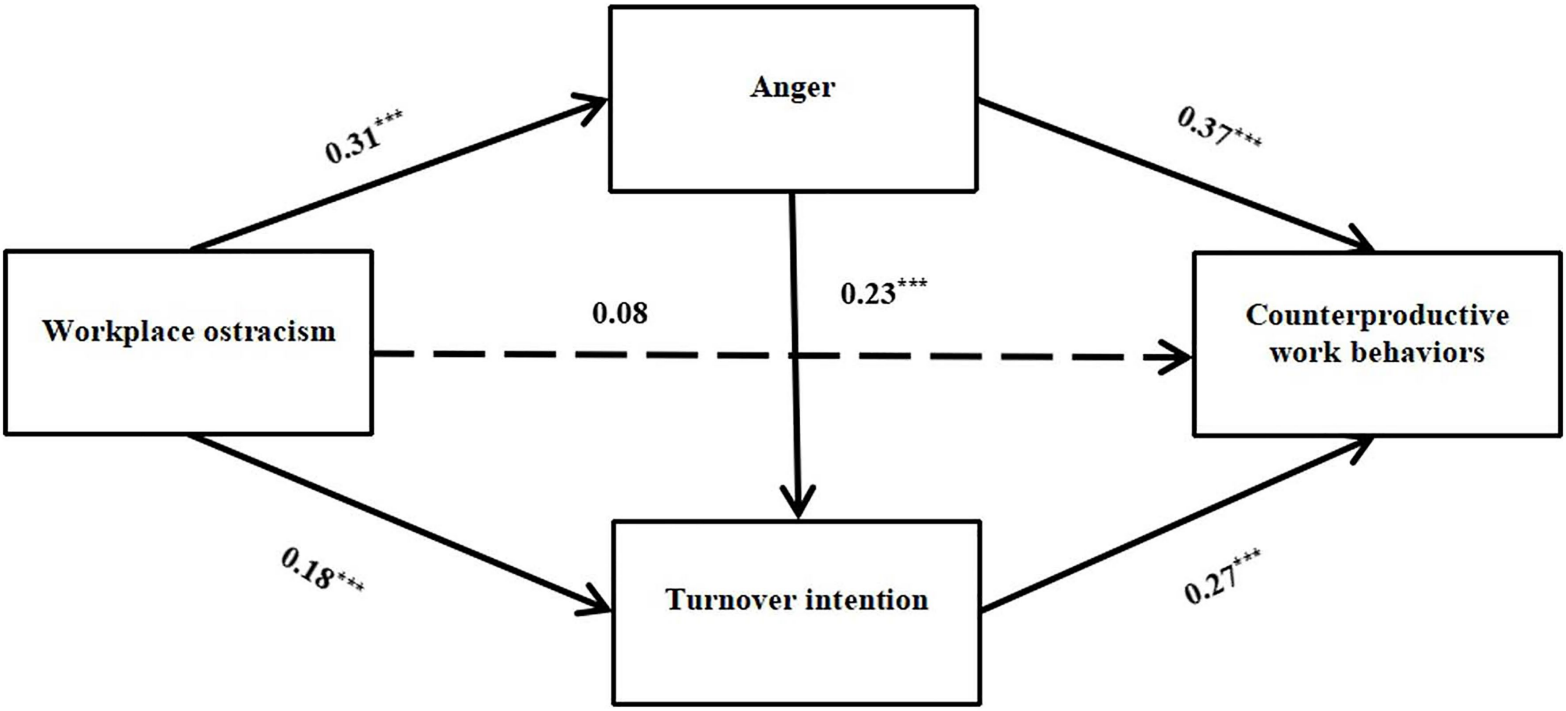
Mediating effect of anger and turnover intention on the relationship between workplace ostracism and counterproductive work behaviors. ^***^p<0.001.

We tested a serial mediation model, which consisted of three indirect effects: (a) workplace ostracism increases counterproductive work behaviors *via* anger, (b) workplace ostracism increases counterproductive work behaviors *via* turnover intention, and (c) workplace ostracism increases counterproductive work behaviors *via* anger and turnover intention (Figure 2).

According to the results of the correlation analysis, only gender, position, and working time were significantly related to counterproductive work behaviors. After controlling for the effects of these variables, the results showed a positive effect of workplace ostracism on anger (*β*=0.31, *t*=6.80, *p*<0.001) and a positive effect of workplace ostracism on turnover intention (*β*=0.18, *t*=3.69, *p*<0.001). There was also a positive relationship between anger and turnover intention (*β*=0.23, *t*=4.77, *p*<0.001). Both anger and turnover intention significantly predicted counterproductive work behaviors (*β*=0.37, *t*=8.35, *p*<0.001, for anger; *β*=0.27, *t*=6.36, *p*<0.001, for turnover intention). The direct effect of workplace ostracism on counterproductive work behaviors was not significant (*β*=0.08, *t*=1.75, *p*>0.05). The results are shown in Table 2.

**Table 2:** Regression analysis of the mediation model.

Predictive variable	Anger			Turnover intention			Counterproductive work behaviors		
	*β*	*SE*	*t*	*β*	*SE*	*t*	*β*	*SE*	*t*
Gender	−0.01	0.05	−0.20	−0.0002	0.05	−0.005	−0.06	0.04	−1.45
Position level	0.08	0.05	1.69	−0.09	0.05	−1.95	0.07	0.04	1.69
Working time	0.10	0.05	2.20^*^	0.07	0.05	1.45	0.02	0.04	0.50
Workplace ostracism	0.31	0.05	6.80^***^	0.18	0.05	3.69^***^	0.08	0.04	1.75
Anger				0.23	0.05	4.77^***^	0.37	0.04	8.35^***^
Turnover intention							0.27	0.04	6.36^***^
*R* ^2^	0.13			0.12			0.33		
*F*	15.20^***^			11.68^***^			34.01^***^		

* *p*<0.05;

and

*** *p*<0.001.

The indirect effect of workplace ostracism on counterproductive work behaviors through anger was significant (*β*=0.12, *SE*=0.02, 95% CI [0.08, 0.17]). This mediation effect (workplace ostracism → anger → counterproductive work behaviors) accounted for 44.32% of the total effect. In addition, turnover intention mediated the relationship between workplace ostracism and counterproductive work behaviors (*β*=0.05, *SE*=0.02, 95% CI [0.02, 0.09]). This mediation effect (workplace ostracism → turnover intention → counterproductive work behaviors) accounted for 18.80% of the total effect. Finally, the indirect effect of workplace ostracism on counterproductive work behaviors through anger and turnover intention (i.e., a chain mediating effect) was also found (*β*=0.02, *SE*=0.01, 95% CI [0.01, 0.04]). This mediation effect (workplace ostracism → anger → turnover intention → counterproductive work behaviors) accounted for 7.64% of the total effect. The results are shown in Table 3.

**Table 3:** Mediating effect analysis of anger and turnover intention on the relationship between workplace ostracism and counterproductive work behaviors.

	Effect	Boot *SE*	95% CI	Relative mediating effect
Total mediation effect	0.18	0.03	[0.14,0.24]	70.76%
Workplace ostracism → anger → counterproductive work behaviors	0.12	0.02	[0.08,0.17]	44.32%
Workplace ostracism → turnover intention → counterproductive work behaviors	0.05	0.02	[0.02,0.09]	18.80%
Workplace ostracism → anger → turnover intention → counterproductive work behaviors	0.02	0.01	[0.01,0.04]	7.64%

Since the three indirect effects were statistically significant, we examined whether these effects were significantly different in terms of their mediating effects. The results showed that the mediating effect of anger was stronger than the mediating effect of turnover intention (*β*=0.07, *SE*=0.03, 95% CI [0.01, 0.13]). Similarly, the mediating effect of anger was stronger than the chain mediating effect of anger and turnover intention (*β*=0.10, *SE*=0.02, 95% CI [0.06, 0.14]). However, there was no significant difference between the mediating effect of turnover intention and the chain mediating effect of anger and turnover intention (*β*=0.03, *SE*=0.02, 95% CI [−0.003, 0.06]).

## Discussion

Based on the social change theory (Li et al., 2019b), this study discussed the influence of workplace ostracism on counterproductive work behaviors and explored how this relationship is mediated by anger and turnover intention.

The results of correlation analysis showed that there was a significant positive correlation between workplace ostracism and anger, turnover intention, and counterproductive work behaviors. This result supports the findings of previous research on workplace ostracism and employee behavior (Chung and Yang, 2017). It also shows that workplace ostracism can negatively impact an organization, as it can affect the emotional states, psychological tendencies, and organizational behaviors of employees.

The results of the mediation analysis showed that workplace ostracism cannot directly predict counterproductive work behaviors; however, it can influence counterproductive work behaviors through the independent and chain mediation of anger and turnover intention. This result verifies the hypothesis of previous studies that workplace ostracism creates a psychological state that encourages counterproductive work behaviors, and its influence on counterproductive work behaviors typically needs to be realized through the mediation of other factors (Wang et al., 2012). Specifically, workplace ostracism can induce anger, leading to counterproductive work behaviors. According to the general strain theory, when an individual experiences a stressful event or situation, he or she will experience one or more negative emotions that induce non-adaptive behaviors (Agnew, 1992). Anger is the most important negative emotion that leads to deviant behavior. When an individual experiences anger due to stressful events, the feeling of injury is enhanced, leading to reduced control. This will easily result in a desire for revenge, thus driving aggressive behaviors (Yang, 2003). Therefore, when individuals perceive that they are excluded they are more prone to anger. Driven by this anger, they use counterproductive work behaviors to relieve their emotions.

Workplace ostracism can also induce counterproductive work behaviors by increasing employees’ turnover intentions. When individuals perceive rejection from other people in the organization they gradually feel that they have less value and significance within the organization, leading to their tendency to leave the organization to find a new place to belong (Singh and Srivastava, 2021). Based on the power dependence theory, as employees decide to leave an organization, they gain a stronger sense of power and feel they have the freedom to choose how to retaliate against workplace ostracism (Tepper et al., 2009). Therefore, the possibility of retaliating against organizations or individuals through counterproductive work behaviors increases in the face of workplace ostracism.

The results also revealed that anger and turnover intention have a chain mediating effect on the relationship between workplace ostracism and counterproductive work behaviors. This result verified the theory of emotional events and showed that emotions and attitudes can have a chain mediating effect on the relationship between events and behaviors (Weiss and Cropanzano, 1996). Workplace ostracism can not only directly affect employees’ behaviors through emotion but also indirectly drive their behaviors through the effect of emotion on their attitudes and tendencies. Compared to general employees, excluded employees may experience more anger, and the cumulative effect will lead to an increasingly strong willingness to leave. Consequently their commitment to the organization will be reduced, eventually leading to counterproductive work behaviors.

This study applies the GAM to situations involving workplace ostracism in order to expand its scope of application. Further, this study focuses on young employees. It is imperative to deepen our understanding of the young to promote the healthy development of this group and their organizations. In this study, the mediating effect of anger was the highest among the three influencing mechanisms. This result confirms the view that the counterproductive work behaviors of young employees are largely influenced by improper emotional venting (Tu, 2016). These employees have insufficient control over their emotions and attitudes and may be impulsive (Zhang et al., 2019c); thus, they tend to engage in extreme behaviors when faced with negative situations. In light of this finding, managers should actively guide employees to realize the importance of positive relationships and the harm of ostracism, while striving to create a good work environment. This action could reduce the occurrence of bad emotions and turnover intention in employees. Simultaneously, organizations should innovate the management methods to young employees according to their ideologies, behavioral preferences, and psychological needs. Organizations should carefully design systems to encourage the emotional management and career growth of employees, and encourage frank and open communication within the organization. These steps will allow organizations to better understand the psychology and work dynamics of their employees, leading to increased care for young employees. By observing their emotional state and behavioral performance, corresponding measures can be implemented to keep them in a positive and peaceful state of mind and reduce impulsive and extreme behaviors.

Our research has some limitations, which we believe can be addressed in future studies. First, this study only paid attention to the young employees born after 1990, and it is not clear whether this mechanism is also applicable to employees from other generations. Future research can further expand the scope of participants to increase the external validity of the study. Second, this study collected self-report data. Although the questionnaire has good reliability and validity, it may lead to homologous errors in data sources because of the lack of a matching survey. Additionally, this study primarily focuses on the negative behaviors and attitudes of employees. Previous studies indicated that people tend to show positive responses to social praise when being investigated (Han and Ren, 2002), which may have an impact on the objectivity of data. Future research may consider selecting multiple information providers and methods to capture the complex mechanism of counterproductive work behaviors. In addition, this study only discusses the effect of anger and turnover intention on counterproductive work behaviors; that is, it only focuses on the influencing factors of counterproductive work behaviors from the emotional and attitude levels based on the GAM. Zhang et al. (2018b) summarized previous studies and proposed that individual differences, interactions between people, and interactions between people and situations can lead to employees’ counterproductive work behaviors. Therefore, future research should use a trait-based approach, as well as social exchange theory and social cognitive theory. This will allow for the exploration of the antecedents of counterproductive work behaviors from the perspectives of demographic variables and trait factors, attitude and perception factors, and situation and work factors.

## Data Availability Statement

The raw data supporting the conclusions of this article will be made available by the authors, without undue reservation.

## Ethics Statement

The studies involving human participants were reviewed and approved by the Research Ethics Committee of Renmin University of China. The patients/participants provided their written informed consent to participate in this study.

## Author Contributions

YZ contributed to the conception and design and drafting the article. DZ and YZ contributed to the collection, analysis, and interpretation of data. DZ contributed to revising the article critically. All authors contributed to the article and approved the submitted version.

## Funding

This research was supported by Major Innovation & Planning Interdisciplinary Platform for the “Double-First” Initiative, Renmin University of China.

## Conflict of Interest

The authors declare that the research was conducted in the absence of any commercial or financial relationships that could be construed as a potential conflict of interest.

## Publisher’s Note

All claims expressed in this article are solely those of the authors and do not necessarily represent those of their affiliated organizations, or those of the publisher, the editors and the reviewers. Any product that may be evaluated in this article, or claim that may be made by its manufacturer, is not guaranteed or endorsed by the publisher.
